# Infrared‐A Irradiation‐induced Inhibition of Human Keratinocyte Proliferation and Potential Mechanisms

**DOI:** 10.1111/php.13248

**Published:** 2020-04-29

**Authors:** Syota Shimizu, Akihiro Aoki, Takuya Takahashi, Fumiki Harano

**Affiliations:** ^1^ Nutraceuticals Division Otsu Skin Care Research Institute Otsuka Pharmaceutical Co., Ltd. Otsu Japan

## Abstract

Infrared‐A (IRA), which can penetrate deeply into the human skin, is a major component of solar radiation and is recognized to promote photoaging of human dermis. To our knowledge, however, the cellular and molecular consequences of human epidermis exposure to IRA have not been clarified. Thus, we investigated whether IRA inhibits the proliferation of normal human epidermal keratinocytes (NHEKs). IRA irradiation ed in cell cycle arrest at G1 and a dose‐dependent reduction in the proliferation of NHEKs. We found that mechanistic target of rapamycin complex 1 (mTORC1) was initially inactivated during IRA irradiation due to the formation of stress granules (SGs), and this inactivation was maintained for at least 6 h after irradiation due to Akt dephosphorylation. Furthermore, repeated exposure of human skin equivalents to IRA led to marked thinning of the epidermal cell layer. In conclusion, IRA irradiation inhibits mTORC1 activity possibly through two molecular mechanisms involving SG formation in the early‐phase and subsequent Akt dephosphorylation. This sequential mechanism seems to cause G1 cell cycle arrest and a reduction in cell proliferation, supporting the hypothesis that the decreased proliferation of basal keratinocytes that occurs during skin aging might be partly attributable to IRA radiation.

## Graphical abstract

IRA radiation is known to cause dermal photoaging in human skin; however, the cellular and molecular effects in human epidermis resulting from IRA exposure have not been clarified. This study demonstrates that IRA inhibits the activity of mTOR complex 1 (mTORC1) in human epidermal keratinocytes and thereby inhibits cell proliferation. We propose that mTORC1 inactivation caused by IRA irradiation proceeds through a sequential mechanism consisting of the early formation of SGs, followed by sustained dephosphorylation of Akt, leading to cell cycle arrest at G1 and a subsequent reduction of cell proliferation.

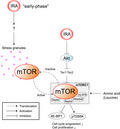

## Introduction

Solar radiation is essential for life on Earth; however, it is also known to cause photoaging of human skin. The spectrum of the sunlight reaching the Earth’s surface ranges from 290 to 3000 nm, with ultraviolet (UV; UVB, 290–320 nm; UVA, 320–400 nm), visible light (400–760 nm) and infrared (IR; IRA, 760–1400 nm; IRB, 1400–3000 nm; IRC, 3000 nm–1 mm) rays accounting for 6.8%, 38.9% and 54.3%, respectively, of incident sunlight (1).

Infrared radiation, which is known to increase the synthesis and expression of matrix metalloproteinase‐1 (MMP‐1) in human dermal fibroblasts through the production of reactive oxygen species (ROS), is thought to play a role in wrinkle formation by enhancing the degradation of type I collagen (2, 3, 4, 5). Furthermore, IR has also been reported to alter the expression of type I collagen (3), and tropoelastin and fibrillin‐1 (6), and to induce angiogenesis (7) and inflammation (8). These physiological and pathological changes that occur in response to IR radiation lead to the abnormal degradation and accumulation of extracellular matrix components, resulting in dermal photoaging. Given that IRA is the predominant source of radiant energy within the terrestrial IR spectrum, it is likely to play a key role in the pathogenesis of photoaging in the human dermis (9, 10, 11, 12). While the evidence for the involvement of IRA in dermal photoaging is increasing, however, little is known about its physiological effects on the human epidermis.

The human epidermis is a self‐renewing multilayered epithelium composed of four layers—the basal layer, spinous layer, granular layer and stratum corneum. Basal keratinocytes can proliferate and generate cells that undergo terminal differentiation. This ultimately leads to the formation of flattened anucleated cornified cells that constitute the stratum corneum, and subsequently desquamate. The above process is called “epidermal turnover”, and there is a close correlation between basal keratinocyte proliferation and the epidermal turnover rate (13). Epidermal turnover has been shown to be slower in the facial skin of older subjects who have received more cumulative life‐time exposure to sunlight as compared with younger subjects (14). That observation may be partly due to the effects of intrinsic aging; nevertheless, it also suggests that the progression of photoaging affects the machinery regulating keratinocyte proliferation. Although a single dose of IRA irradiation suppresses the proliferation of mouse and guinea pig keratinocytes (15, 16), its effect on the human epidermis has not previously been examined.

The evolutionarily conserved mechanistic target of rapamycin (mTOR) is a serine/threonine kinase that plays a key role in regulating cell proliferation in the skin and other organs (17, 18). mTOR exists as two types of complexes, namely rapamycin‐sensitive mTORC1 and rapamycin‐insensitive mTORC2, which differ in protein composition, downstream targets and biological effects. mTORC1, which comprises the protein kinase mTOR and several associated proteins, regulates mRNA translation, cell cycle progression and cell proliferation through the direct phosphorylation of eukaryotic translation initiation factor 4E‐binding protein 1 (4E‐BP1) and S6 kinase (p70S6K) (19). Thus, cellular phosphorylation states of 4E‐BP1 and p70S6K have been used as indicators of mTORC1 activity, and their dephosphorylation has been recognized to contribute to inhibition of cell proliferation (20).

Upstream of mTORC1, growth factor stimulation leads to activation of phosphoinositide 3‐kinase (PI3K) and its downstream molecule Akt, triggering activation of mTORC1 (20, 21), whereas energy stress and the tumor suppressor Lkb1 inhibit mTORC1‐mediated signaling through the AMP‐activated protein kinase (19, 22). Recently, it has been shown that the formation of discrete cytoplasmic foci called stress granules (SGs) in response to various stress conditions, such as oxidative or heat stress, is also associated with inactivation of mTORC1 (23, 24). Because the biological effects of IR are ascribed to enhanced oxidative and heat stress in human skin tissue (3, 4, 7, 25), it is possible that IRA also acts as an environmental factor that damages the skin, leading to mTORC1 inactivation in human skin keratinocytes.

Elucidating the molecular mechanism underlying the effects of IRA irradiation on epidermal cell proliferation might contribute to our understanding of cutaneous photoaging and thereby facilitate the development of more effective strategies to prevent this process. Thus, the aim of this work was to investigate the potential antiproliferative effects of IRA on human epidermal keratinocytes at the cellular and molecular level.

## Materials and methods

### Cell culture and skin equivalent model

NHEKs (Lifeline Cell Technology, Frederick, MD, USA) isolated from newborn foreskin were cultured in Humedia‐KG2 (Kurabo Biomedicals, Osaka, Japan) at 37°C with 5% CO_2_. EpiDerm‐FT™ full‐thickness human skin equivalents (EFT‐400) and maintenance medium (EFT‐400‐ASY) were obtained from MatTek Corporation (Ashland, MA, USA). Upon receipt, the skin equivalents were immediately placed in 6‐well plates containing 2.5 mL of EFT‐400‐ASY and maintained at 37°C with 5% CO_2_. The skin equivalents were used the following day.

### IRA irradiation

To obtain IRA in the 760–1400 nm range, we used IR lamps (IR100/110V100WR; Toshiba, Tokyo, Japan) equipped with a long‐pass filter (IR70; Mitsubishi Chemical, Tokyo, Japan) and water filter. Prior to IRA irradiation, NHEKs were incubated in Humedia‐KB2 (Kurabo Biomedicals) for 24 h at 37°C with 5% CO_2_. NHEKs and skin equivalents were exposed to IRA with an irradiance of 80 mW cm^−2^ at doses ranging from 100 J cm^−2^ (21 min) to 800 J cm^−2^ (167 min). During exposure, the cells and skin equivalents were maintained in Hank’s balanced salt solution (Thermo Fisher Scientific, Rockford, IL, USA) at approximately 30°C. Accordingly, non‐irradiated control cells were kept in an incubator at 30°C for the duration of the irradiation.

### Proliferation assay

NHEKs were seeded at 50,000 cells in 35‐mm dishes and exposed to different doses of IRA (200–800 J cm^−2^). After IRA irradiation, the culture media was replaced with Humedia‐KB2, and the cells were incubated at 37°C with 5% CO_2_. Cell proliferation was determined at 24, 48 and 72 h after IRA irradiation by directly counting the cells using an automated cell counter.

### Detection of lactate dehydrogenase (LDH) release

Release of LDH by cells was measured at 48 h after IRA irradiation (800 J cm^−2^) by using the LDH Cytotoxicity Detection Kit (Takara Bio Inc., Shiga, Japan). The percentage of LDH release was calculated as follows: Percentage release = 100 × (experimental LDH release – background of medium)/(maximal LDH release − background of medium). To determine the maximal LDH release, NHEKs were treated with 1% Triton™ X‐100 for 2 h at 37°C.

### Flow cytometry

Cell cycle distribution was analyzed at 24 h after IRA irradiation (800 J cm^−2^) by using the BrdU Flow Kit (BD Biosciences, San Jose, CA, USA). NHEKs were trypsinized, fixed, stained with 7‐aminoactinomycin D and analyzed by using a FACSVerse™ flow cytometer (BD Biosciences) and ModFit LT™ software (Verity Software House, Topsham, ME, USA) to determine the cell cycle distribution.

### Leucine and cycloheximide (CHX) treatments

Immediately after IRA irradiation (400 J cm^−2^), cells were treated with or without 20 μM CHX (Abcam, Cambridge, UK) for 5 min followed by 1 mM leucine for 20 min at 37°C with 5% CO_2_.

### Western blot analysis

NHEKs were lysed in lysis buffer (Thermo Fisher Scientific). After quantification, equal amounts of protein were subjected to gel electrophoresis on 4%–15% sodium dodecyl sulfate/polyacrylamide gels and blotted onto polyvinylidene fluoride membranes. Subsequently, membranes were blocked with 5% non‐fat dried milk in TBS/T (Cell Signaling Technology, Danvers, MA, USA) for 1 h at room temperature and incubated with the appropriate antibodies at 4°C overnight. Signals were detected with an enhanced chemiluminescence detection kit (Cell Signaling Technology). Primary antibodies for pan‐Akt (#4691, 1:1000), phospho‐Akt (Thr308) (#13038, 1:1000), phospho‐Akt (Ser473) (#4060, 1:2000), mTOR (#2983, 1:1000), 4E‐BP1 (#9644, 1:1000), phospho‐4E‐BP1 (Ser65) (#13443, 1:1,000), p70S6K (#2708 1:1000), phospho‐p70S6K (Thr389) (#9234, 1:1000), cyclin D1 (#2978, 1:1000), cyclin D3 (#2936, 1:1000), CDK4 (#12790, 1:1000), CDK6 (#13331, 1:1000), caspase‐3 (#9662, 1:1000) and β‐actin (#4970, 1:1000) were purchased from Cell Signaling Technology.

### Immunofluorescence microscopy

Immediately after exposure to different doses of IRA (200–800 J cm^−2^), NHEKs were fixed with 4% paraformaldehyde followed by methanol at − 20°C, and then incubated with blocking reagent for 1 h at room temperature. The cells were then treated with anti‐mTOR antibody (Cell Signaling Technology, #2983, 1:400) at 4°C overnight, followed by anti‐Ras GTPase‐activating protein binding protein 1 (G3BP) antibody (Abcam, ab56574, 1:500) for 1 h at room temperature. Fluorescence images were acquired at × 40 magnification using a fluorescence microscope (BZ‐X700; Keyence, Osaka, Japan).

### Immunohistochemistry

Skin equivalents were irradiated with IRA (800 J cm^−2^ day^−1^) for up to 5 consecutive days. Each tissue was fixed with 10% formalin and embedded in paraffin, and cross‐sections (5–7μm thick) were stained with hematoxylin‐eosin to measure the thicknesses of the epidermal cell layer and stratum corneum. The epidermal cell layer was defined as the distance from the top of the granular layer to the bottom of the basal layer. To examine the expression of Ki‐67, specimens were treated with anti‐Ki‐67 antibody (Cell Signaling Technology, #9449, 1:400) for 1 h at room temperature. The number of Ki‐67‐positive cells in the microscopic field at × 10 magnification of each tissue section was counted (5 sections per group). Bright field images were acquired using a fluorescence microscope (BZ‐X700; Keyence).

### Statistical analysis

Data were analyzed by using SAS software version 9.3 (SAS Institute, Cary, NC, USA). A *P*‐value < 0.05 was considered statistically significant. The statistical analysis methods used in each experiment are summarized in the figure legends.

## Results

### IRA irradiation inhibits the proliferation of NHEKs

**Figure 1:**
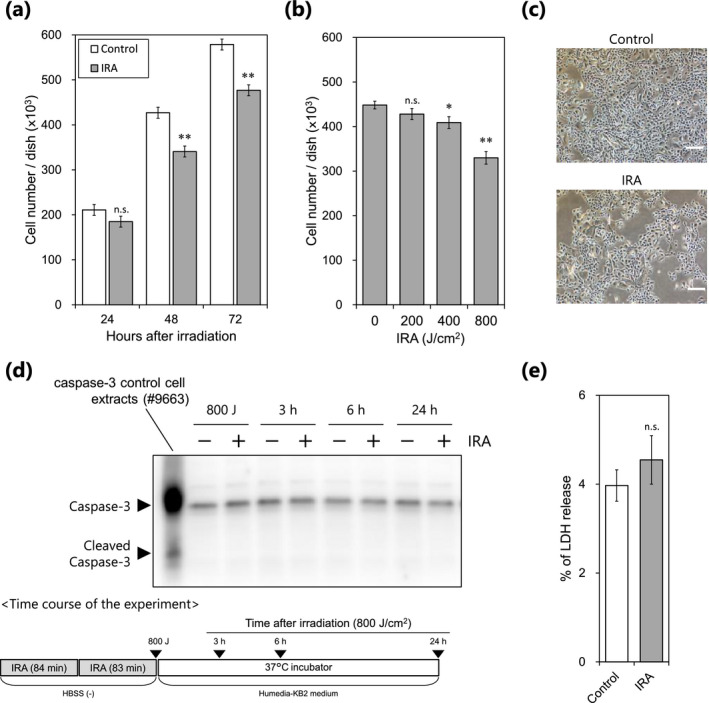
Effects of IRA irradiation on cell proliferation of NHEKs. (a) Cells were counted at the indicated time points after IRA irradiation (800 J cm^−2^). Data are the mean ± standard error (SE) of four independent experiments (*n* = 4). **P < 0.01 *vs* control by three‐way ANOVA (group–time interaction at each time). (b) Cells were counted 48 h after IRA irradiation (200–800 J cm^−2^). Data are the mean ± SE of two independent experiments (*n* = 10). IRA dose‐dependently reduced the number of NHEKs by Williams’ test (*P < 0.05, **P < 0.01 *vs* control). (c) Representative images of control and IRA‐irradiated cells captured 48 h after IRA irradiation (800 J cm^−2^). Scale bar represents 100 μm. (d) Western blot analysis was performed at the indicated time points (▼) after IRA irradiation (800 J cm^−2^). Representative results from three independent experiments are shown. (e) LDH release was examined 48 h after IRA irradiation (800 J cm^−2^). Data are the mean ± SE of three independent experiments (*n* = 12). There was no significant difference between control and IRA‐irradiated cells by two‐way ANOVA (Tukey). n.s., not significant.

We confirmed that humans may be exposed to up to ~ 800 J cm^−2^ of IRA radiation from sunlight between 9 am and 5 pm on a typical day in August 2012 in Shiga, Japan (data not shown). In this study, therefore, we conducted dose escalation tests on NHEKs up to a maximum dose of 800 J cm^−2^ of IRA in one exposure. We found that the number of NHEKs was significantly reduced at 48 and 72 h post‐irradiation (Fig. 1a), and the extent of the reduction was dose‐dependent (Fig. 1b). Cell morphological changes (Fig. 1c) and activated caspase‐3 (Fig. 1d), a marker of mammalian/human cells undergoing apoptosis, were not observed after IRA irradiation. Moreover, there was no significant cytotoxicity by LDH assay (Fig. 1e). These results indicate that the IRA‐induced decrease in cell numbers of NHEKs is caused by an antiproliferative effect rather than a cytotoxic effect.

### Effects of IRA irradiation on cell cycle progression

**Figure 2:**
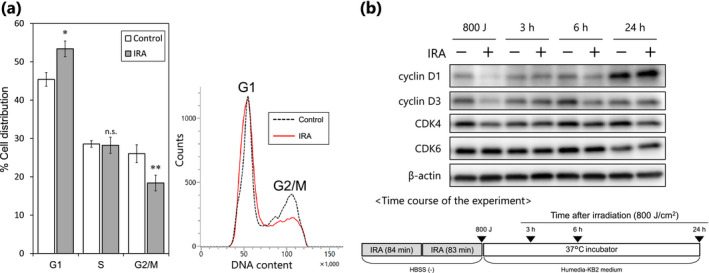
IRA irradiation causes cell cycle arrest at the G1 phase in NHEKs. (a) Shown are quantification (left) and representative flow‐cytometric histograms (right) of cell cycle distribution analysis performed 24 h after IRA irradiation (800 J cm^−2^). Data are the mean ± SE of four independent experiments (*n* = 4). **P < 0.01 *vs* control by unpaired two‐tailed Student’s *t*‐test. (b) Western blot analysis was performed at the indicated time points (▼) after IRA irradiation (800 J cm^−2^). Representative results from three independent experiments are shown.

Based on the above results, we determined whether inhibition of cell proliferation after IRA irradiation is associated with the effects of IRA on cell cycle regulation. Analysis of the cell cycle distribution indicated that IRA irradiation significantly arrested the cell cycle at G1, with a concomitant reduction in the number of cells in G2/M, but it did not alter the number of cells in S phase (Fig. 2a).

Progression through the G1 phase of the cell cycle is principally regulated by the cyclin D and cyclin‐dependent kinase (CDK) 4/6 complexes. To explore the molecular mechanisms involved in IRA‐induced G1 cell cycle arrest, we exposed cells to IRA and analyzed protein samples by Western blot. As shown in Fig. 2b, IRA exposure resulted in a reduction in the expression levels of cyclin D1, D3, and CDK4, whereas the level of CDK6 was not altered after IRA exposure. These results suggest that the IRA‐induced reduction in cell proliferation may be associated with the induction of G1 cell cycle arrest, which might be caused by suppressed levels of cyclin D and CDK4 in NHEKs.

### IRA irradiation inhibits Akt/mTORC1 activity

**Figure 3:**
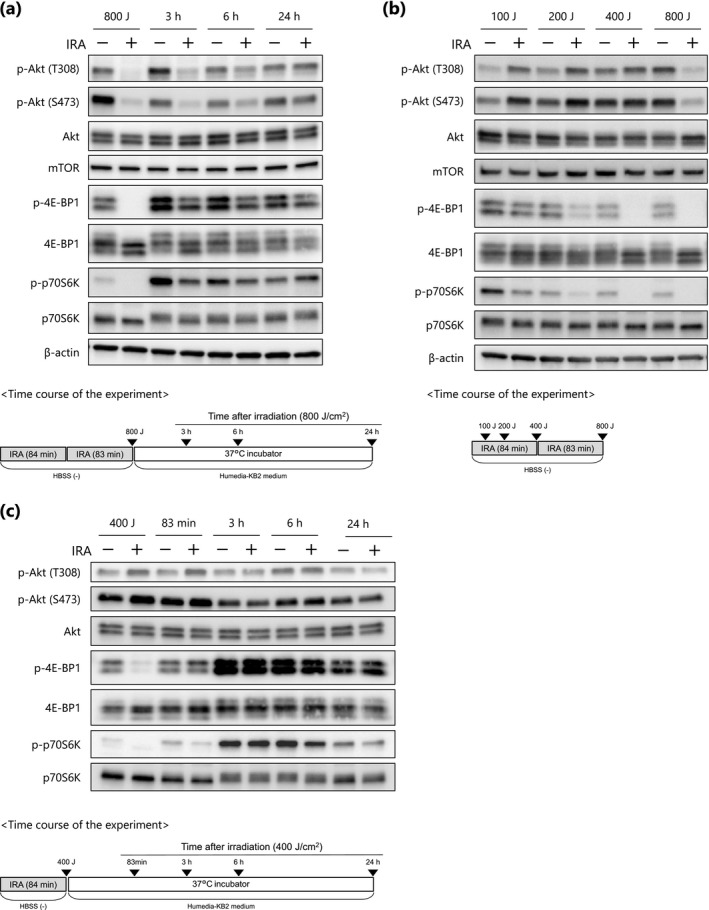
IRA irradiation modulates the Akt/mTORC1 signaling pathway. (a–c) NHEKs were irradiated with IRA at 800 J cm^−2^ (a), doses up to 800 J cm^−2^ (b) or 400 J cm^−2^ (c), and Western blot analysis was performed at the indicated time points (▼). Representative results from three independent experiments are shown.

To investigate the signaling pathways by which IRA inhibits NHEK proliferation, we assessed the phosphorylation status of proteins within the Akt/mTORC1 pathway by Western blotting. Phosphorylation of Akt at Thr308 and Ser473 was significantly attenuated after exposure to 800 J cm^−2^ of IRA, and this reduction remained for at least 6 h post‐irradiation (Fig. 3a). After that, the phosphorylation state returned to basal levels at 24 h post‐irradiation. In addition, two of the immediate downstream targets of mTORC1, 4E‐BP1 and p70S6K, were dephosphorylated to coincide temporally with Akt dephosphorylation after IRA irradiation (800 J cm^−2^), suggesting that inhibition of mTORC1 is strongly correlated with Akt activity. Collectively, these findings suggest that Akt is probably an important target of IRA in the inactivation of mTORC1.

Intriguingly, we also noticed that at time points corresponding to a dose of 200–400 J cm^−2^ of IRA, the phosphorylation of 4E‐BP1 and p70S6K was markedly decreased even though Akt activity was not reduced (Fig. 3b). Rather, low doses of IRA (100–200 J cm^−2^) suppressed phosphorylation of 4E‐BP1 and p70S6k, and simultaneously induced activation of Akt (Fig. 3b, lanes 2 and 4). This result seems to be consistent with a previous study showing that p70S6K activity negatively regulates Akt phosphorylation and that treatment with the mTOR inhibitor rapamycin increases the phosphorylation of Akt via inhibition of p70S6K (26). In addition, we confirmed that IRA exposure (400 J cm^−2^) had no inhibitory effect on Akt activity until 24 h post‐irradiation (Fig. 3c), suggesting that the inhibition of mTORC1 that occurred within the dose range of 200 to 400 J cm^−2^ is independent of the mechanism mediated by Akt dephosphorylation. Note that, in control cells, the gradual decline in phosphorylation of 4E‐BP1 and p70S6K during IRA irradiation (Fig. 3b, lanes 1, 3, 5 and 7) and the marked increase at 3 and 6 h post‐irradiation (Fig. 3a, lanes 3 and 5) are likely to be caused by amino acid depletion and resupply, respectively (27, 28). Because the cells were maintained in HBSS (‐) with no amino acids during IRA irradiation, it is conceivable that amino acids might have been depleted in these cells. Taken together, the results shown in Fig. 3 suggest that inactivation of mTORC1 by IRA may occur through at least two distinct molecular mechanisms, one Akt‐independent and one Akt‐mediated, based on exposure doses of 200–400 (termed “early‐phase”) and 800 J cm^−2^ respectively.

### IRA irradiation induces sequestration of mTOR into SGs

**Figure 4:**
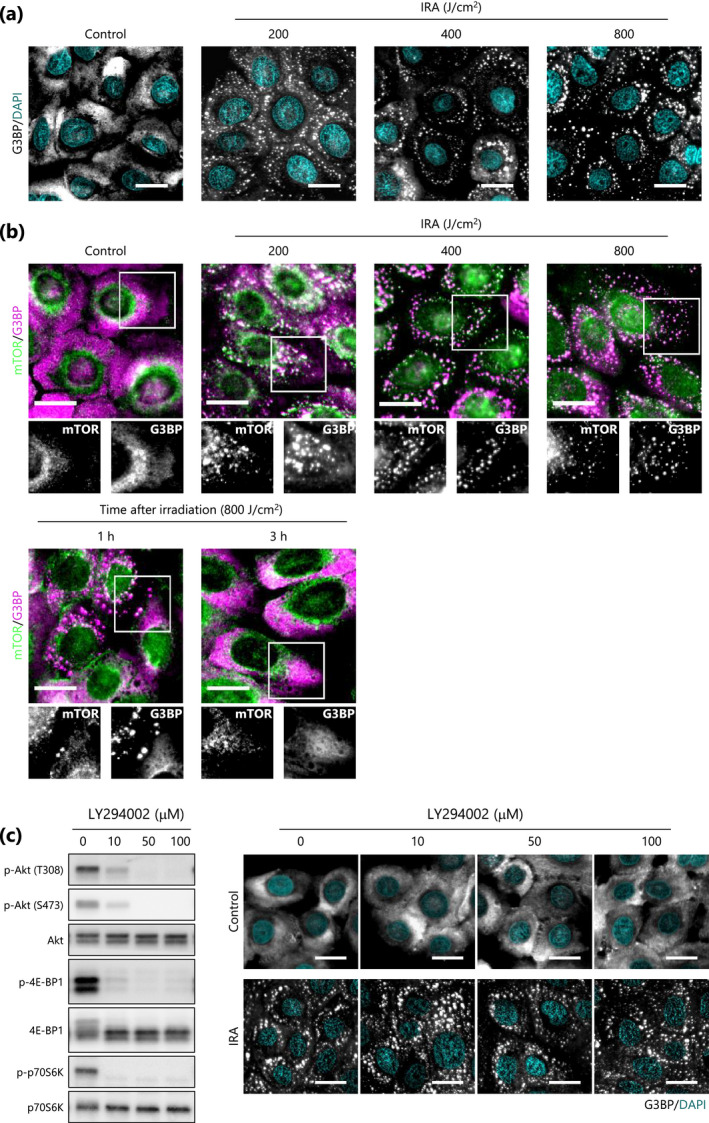
IRA irradiation induces sequestration of mTOR into SGs in NHEKs. (a,b) Representative immunofluorescence images of SG formation (a) and colocalization between SGs and mTOR (b) were acquired at the indicated time points after IRA irradiation (200–800 J cm^−2^). (c) Representative Western blot analysis was performed after treatment of NHEKs with LY294002 (10–100 μM) for 1 h (left), and then immunofluorescence images were acquired with or without IRA irradiation (400 J cm^−2^) (right). Scale bar represents 20 μm.

Sequestration of mTOR into SGs causes inactivation of mTORC1 in mammalian cells (24); we thus hypothesized that IRA might act as a potent stimulus for SG formation, leading to mTORC1 inactivation in an Akt‐independent manner in NHEKs. As expected, immunofluorescent staining showed that G3BP, a stress granule‐specific marker (29), formed discrete cytoplasmic foci (i.e. SGs) upon IRA irradiation at a dose of at least 200 J cm^−2^ (Fig. 4a), and the SGs appeared to be colocalized with mTOR (Fig. 4b, upper). Subsequently, this colocalization was markedly decreased within 1 h post‐irradiation and had completely disappeared at 3 h, with the proteins distributed diffusely throughout the cytoplasm (Fig. 4b, lower). This observation is consistent with previous research showing that the formation of SGs due to oxidative and osmotic stress can be transient (30, 31) and suggests that IRA‐induced SG formation is also reversible and transient. Furthermore, treatment with the PI3K/Akt inhibitor LY294002 led to marked inhibition of Akt but affected neither SG formation in non‐irradiated cells nor IRA‐induced SG formation, confirming that Akt is not involved in this process (Fig. 4c). Taken together, our findings suggest that IRA‐induced sequestration of mTOR into SGs is a possible mechanism for early‐phase inactivation of mTORC1.

### Early‐phase inactivation of mTORC1 is SG‐dependent

**Figure 5:**
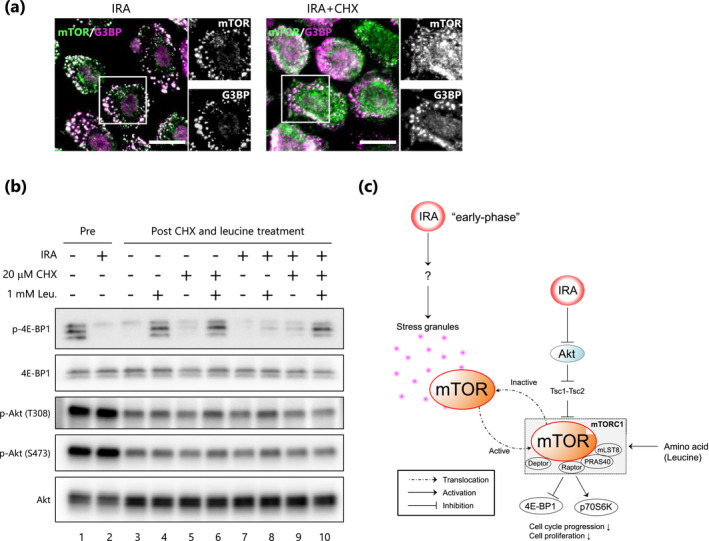
Sequestration of mTOR into SGs upon IRA irradiation is responsible for mTORC1 inactivation. (a) Representative immunofluorescence images showing colocalization between SGs and mTOR were acquired after IRA irradiation (400 J cm^−2^) with CHX (20 μM) pre‐treatment for 5 min. Scale bar represents 20 μm. (b) After IRA irradiation (400 J cm^−2^), cells were lysed immediately (Pre) or after pre‐treatment with or without CHX (20 μM) followed by 1 mM leucine treatment (Post). Representative results from three experiments are shown. (c) Proposed model of molecular mechanisms involved in the regulation of mTORC1 signaling upon IRA irradiation.

To investigate whether the formation of SGs induced by IRA is responsible for the punctate localization of mTOR, we made use of the SG‐dissolving property of cycloheximide (CHX) (24). Immunofluorescent staining showed that dissolution of IRA‐induced SGs by treatment with CHX resulted in reduced punctate colocalization of the marker G3BP with mTOR (Fig. 5a), indicating that the formation of mTOR foci is dependent on SGs.

Next, in order to determine whether the IRA‐induced SGs exert an inhibitory effect on mTORC1, we used leucine, which is a potent activator of mTORC1 (27, 28). As shown in Fig. 5b, phosphorylation of 4E‐BP1 was transiently impaired even in non‐irradiated control cells due to medium exchange after the irradiation (compare lane 1 with lane 3); subsequently, however, leucine clearly and efficiently phosphorylated 4E‐BP1 (compare lane 3 with lane 4). Under these conditions, IRA irradiation inhibited leucine‐induced phosphorylation of 4E‐BP1 (compare lane 8 with lane 4), whereas post‐treatment with CHX resulted in substantial restoration of the phosphorylation (compare lane 8 with lane 10), indicating that SG formation is responsible for mTORC1 inhibition. Moreover, as described previously (32), leucine treatment did not alter the phosphorylation state of Akt, confirming that this effect was independent of Akt. Thus, we conclude that IRA‐induced SG formation is related to mTORC1 inactivation and this mechanism may contribute to the early‐phase inactivation of mTORC1.

### Effects of IRA irradiation on epidermal morphology in human skin equivalents

**Figure 6:**
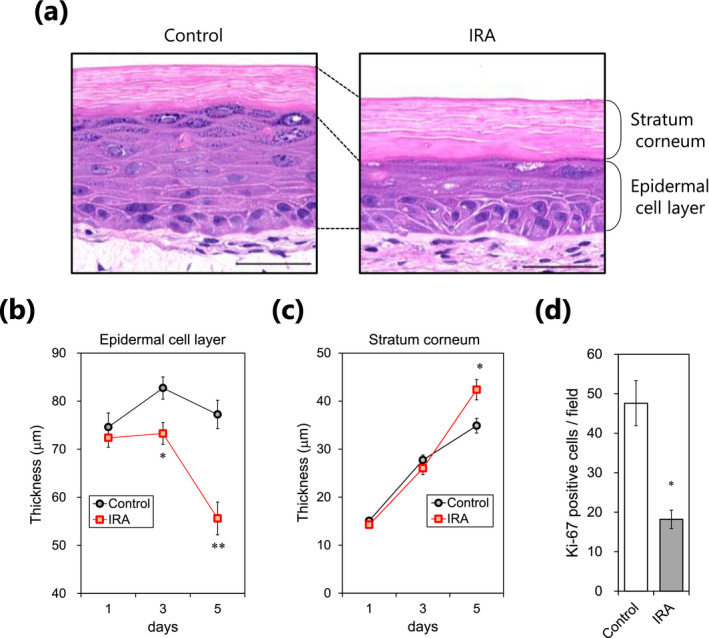
Influence of repeated exposure to IRA on epidermal thickness and suprabasal cell proliferation in human skin equivalents. (a) Representative images of control and IRA‐irradiated groups were acquired on day 5. Scale bar represents 50 μm. (b, c) Quantification of epidermal cell layer and stratum corneum thickness (μm) at the indicated time points. Data are the mean ± SE of five skin equivalents. *P < 0.05, **P < 0.01 *vs* control by unpaired two‐tailed Student’s *t*‐test. (d) The number of Ki‐67‐positive cells was assessed by immunocytochemistry on day 5. Data are the mean ± SE of five skin equivalents. **P < 0.01 *vs* control by unpaired two‐tailed Student’s *t*‐test. Scale bar represents 100 μm.

To further examine the physiological relevance of IRA, we repeatedly exposed human skin equivalents to 800 J cm^−2^ of IRA for up to 5 consecutive days. Histological sections stained with hematoxylin‐eosin revealed that repeated exposure to IRA led to marked differences in epidermal morphology as compared with non‐irradiated control skin equivalents (Fig. 6a). On day 5, the control group showed substantial thickening of the epidermal cell layer, indicative of cell proliferation; by contrast, the IRA‐exposed skin group showed significant attenuation of the thickening, which appeared likely to be due to thinning of the spinous layer (Fig. 6b). Conversely, a significant increase in the thickness of the stratum corneum was observed in the IRA‐irradiated group (Fig. 6c). In addition, the number of Ki‐67‐positive cells in the basal layer was significantly decreased in the IRA‐exposed group (Fig. 6d). These results suggest that repeated exposure to IRA radiation may have a cumulative effect on histological changes in the human epidermis and the changes may be accompanied by the inhibition of basal keratinocyte proliferation.

## Discussion

As human skin ages, keratinocytes in the epidermis become shorter (33), stratum corneum transit time becomes longer (14, 34) and the epidermis becomes thinner (35). These histological and physiological changes are also observed in severely photoaged skin and are thought to be mainly due to the reduced proliferation of epidermal keratinocytes (14, 36, 37). We, therefore, hypothesized that IRA may potentially modulate the proliferation of human keratinocytes for two reasons: first, it can inhibit both DNA synthesis and proliferation of keratinocytes in rodent epidermis (15, 16); second, it may act as a potent stimulus for SG formation, which lead to reduced mTORC1 activity, in human skin (i.e. by generating heat and ROS) (3, 4, 7, 25). To our knowledge, however, previous studies have neither identified the molecular mechanism(s) underlying the inhibitory effect of IRA on cell proliferation nor examined its effect specifically on human keratinocytes. In the present study, therefore, we sought to determine whether IRA radiation exerts an antiproliferative effect on human skin keratinocytes and, if so, to clarify the potential mechanism(s) that might be involved in this effect.

Our study confirms that physiologically relevant doses of IRA have a growth inhibitory effect and induce G1 cell cycle arrest along with marked alterations in the expression of G1‐associated proteins, including cyclin D1, cyclin D3, and CDK4 in NHEKs. It is known that cyclins and CDKs are critical regulators of cell cycle progression. In early G1 phase, cyclin D binds to CDK4/6 to form a cyclin‐CDK complex and subsequently activates the Rb protein, resulting in G1 cell cycle progression (38). In addition, suppression of cyclin D and CDK4 leads to growth arrest in carcinoma cells (39, 40). Hence, our results indicate that inhibition of cyclin D and CDK4 expression leading to G1 cell cycle arrest may be a possible mechanism underlying the antiproliferative effect of IRA in NHEKs.

Rapamycin, a potent inhibitor of mTORC1, and LY294002, an inhibitor of PI3K/Akt, are known to be key negative regulators of cell proliferation via the suppression of cyclin D and CDK4, whereas CDK6 is not significantly associated with this pathway (39). It has also been reported that Akt/mTORC1 plays a central role in cell proliferation by inducing the translation of 5’TOP mRNA transcripts via phosphorylation of 4E‐BP1 and p70S6K (41, 42, 43). Thus, to test the hypothesis that IRA regulates cell proliferation via the Akt/mTORC1 pathway in NHEKs, we examined mTORC1 activation by assessing phosphorylation of 4E‐BP1 and p70S6K as indicators. Western blot analysis revealed that 4E‐BP1 and p70S6K were rapidly dephosphorylated upon exposure to IRA, and this reduction in phosphorylation remained for at least 6 h post‐irradiation. Further investigation indicated that inactivation of mTORC1 proceeds through a sequential mechanism consisting of the formation of SGs in the early‐phase of exposure, followed by Akt dephosphorylation (Fig. 5c). It is interesting to note that rapamycin‐induced complete G1 cell cycle arrest requires the inhibition of both 4E‐BP1 and p70S6K phosphorylation, whereas the inhibition of p70S6K alone is insufficient for complete arrest (41). In addition, 4E‐BPs regulate cell cycle progression and cell proliferation independent of p70S6Ks (44). Because those observations indicate that 4E‐BP1 is a critical target of mTORC1 in promoting cell proliferation, we focused on its phosphorylation state in this study.

We found that IRA‐induced SG formation contributed to the early‐phase inactivation of mTORC1. SGs, which form during the stress response, are non‐membranous assemblies of mRNA and messenger ribonucleoprotein where initiation of bulk translation is limited (29). As a consequence, many cellular activities, in addition to mRNA translation, are generally inhibited. Recently, it was reported that, under oxidative and heat stress conditions, SG formation in both yeast and mammalian cells can inhibit TORC1 signaling by sequestering its constituent molecules including mTOR (24), raptor (23) and Kog1 (31), and this effect is not mediated by the PI3K/Akt pathway (24). Accordingly, we observed that SG formation occurred when Akt was constitutively active and showed that colocalization of SGs with mTOR may be responsible for both delaying mTORC1 reactivation by leucine and inhibiting basal mTORC1 activity, similar to a previous report (31). In addition, our findings that IRA exerts no cytotoxicity and IRA‐induced SG formation is transient are supported by a previous study showing that SG formation is reversible in cells that recover from nonlethal stress (30). Taken together, these results reveal an unexpected mechanism for Akt‐independent regulation of mTORC1 activity in the early‐phase of response to IRA and suggest that inhibition predominantly regulated by SG formation may have a considerable role in preventing NHEK proliferation.

To further confirm the relationship between SG formation and cell proliferation, we attempted to evaluate whether the antiproliferative effect of IRA could be rescued by CHX treatment. Unfortunately, however, because of the protein synthesis‐inhibiting effect of CHX (45), this experiment was not feasible because CHX alone showed a significant growth inhibitory effect in NHEKs (data not shown). In addition to CHX, emetine is often used as a drug that degrades SGs (29), but it also has an inhibitory effect on protein synthesis; therefore, we could not perform a proliferation assay to show whether degradation of SGs restores growth inhibition. Because arsenite‐induced oxidative stress is a well‐known inducer of SG formation (23), we hypothesized that, if ROS are involved in the formation of SGs, the exogenous addition of antioxidants such as ascorbic acid may restore cell proliferation via degradation of SGs. Although intracellular ROS have been reported to be important in the cellular response to IRA exposure in fibroblasts (5, 10, 11), it is unlikely that ROS are sufficient to trigger the formation of SGs in NHEKs. Specifically, we found that IRA exposure increased the amount of intracellular ROS in NHEKs and that simultaneous treatment with ascorbic acid effectively prevented this increase; however, no obvious effect on SG formation, or sequestration of mTOR, was observed (see Figure S1). Besides, to exclude the possibility of heat‐related effects, we used a water‐filtered IRA source that generated no heat in order to maintain the temperature of the culture supernatant at around 30°C during the irradiation experiment (9); nevertheless, we cannot rule out the possibility that a local increase in temperature in the cytoplasm might have been involved in the formation of SGs (46). Further work is needed to elucidate the mechanisms by which IRA promotes SG formation.

Our study indicates that the phosphorylation status of Akt following early‐phase inactivation due to SG formation may dictate the duration of mTORC1 inactivation in response to IRA irradiation. We observed that Akt was dephosphorylated at both Thr308 and Ser473, residues that are necessary for maximal kinase activation (47), and this reduction was positively correlated with the diminished mTORC1 activity that occurred after 800 J cm^−2^ of IRA irradiation in NHEKs. Given that the early‐phase inhibition that occurred upon IRA (400 J cm^−2^) without Akt inactivation was transient (Fig. 3c), it is expected that the sustained inhibition of mTORC1 for up to 6 h post‐irradiation (800 J cm^−2^) would be due to Akt dephosphorylation. In addition, IRA exerted a dose‐dependent inhibitory effect against NHEK proliferation, suggesting that the prolonged duration of mTORC1 inactivation owing to the sequential mechanism of SG formation and Akt dephosphorylation may be important for growth suppression. It has been reported that cross‐talk and/or cooperative effects between the PI3K/Akt and MEK/ERK pathways also mediate mTORC1 activity (48); here, however, we found that MEK inhibition by PD98059 resulted in only weak inhibition of p70S6K and no inhibition of 4E‐BP1 phosphorylation (data not shown). Collectively, these data suggest that Akt might play a pivotal role in modulating mTORC1 activity in response to IRA exposure.

We further investigated the mechanisms responsible for the upstream regulation of Akt upon IRA irradiation; however, several questions remain to be answered. Akt is phosphorylated at Thr308 by PDK1, which is absolutely required for its kinase activity (49), and at Ser473 by mTORC2, which further potentiates its activity 4‐ to 5‐fold in cells (47). We also examined the involvement of PDK1 in IRA‐mediated Akt inactivation by assessing its phosphorylation state in NHEKs after IRA exposure; however, no significant changes were observed (data not shown). It seems likely that PDK1 dephosphorylation is not essential for IRA‐mediated Akt inactivation, and the actual signaling molecules responsible for phosphorylation of Thr308 and Ser473 in Akt remain to be elucidated.

Based on previous studies showing that the Akt/mTORC1 signaling pathway is also highly relevant in skin tissue function, including proliferation (18, 50), wound healing (51) and barrier function (18), we hypothesized that IRA irradiation might exert physiological effects in human skin equivalents. We found that repeated IRA exposure caused epidermal atrophy and thickening of the stratum corneum, which was densely compacted, probably reflecting a decrease in keratinocyte proliferation (13, 14, 36). Our findings indicate that the antiproliferative effect of IRA may lead to histological changes similar to those observed in the human epidermis of photoaged skin; however, the precise contribution of IR radiation to human skin aging remains controversial. For instance, photobiomodulation devices with wavelengths from red to near‐IR (formally known as “low‐level laser therapy”) have been shown to be effective for improving photoaged skin, including treating periorbital skin wrinkles (52), promoting wound healing (53) and driving cell proliferation (54). At first glance, this seems contradictory to several studies showing that IR radiation cause photoaging. It is possible that the desired biological responses to laser stimulation are dependent on various device parameters such as wavelength (nm), power density (mW cm^−2^), dose (J cm^−2^) and pulse duration and that these parameters must be meticulously adjusted in order to maximize the desired outcome. These observations suggest that the effects of IR on the skin vary with wavelength and power density, and it is conceivable that exposure to the broadband wavelength and relatively high doses of IR from sunlight is more likely to contribute to photoaging. Further work is needed to elucidate the wavelength‐specific effects of IR on human skin to facilitate the development of more effective strategies to prevent the photoaging process.

In conclusion, we have demonstrated for the first time that physiologically relevant doses of IRA radiation exert an antiproliferative effect due to G1 cell cycle arrest, and this effect is positively correlated with mTORC1 inactivation in human epidermal keratinocytes. We propose that mTORC1 inactivation caused by IRA irradiation proceeds through a sequential mechanism consisting of the early formation of SGs, followed by sustained dephosphorylation of Akt. These results have led us to reconsider the effect of IRA radiation on the human epidermis and support the hypothesis that IRA radiation might partly contribute to the decreased proliferation of basal keratinocytes that occurs during the progression of skin aging.

## Supporting information


**Figure S1.** IRA irradiation causes ROS generation but it may not be involved in SG formation in NHEKs.
