# Supplementary material for: Infrared‐A Irradiation‐induced Inhibition of Human Keratinocyte Proliferation and Potential Mechanisms
Source: Photochem Photobiol. 2020 Apr 29;96(5):1105–15. doi: 10.1111/php.13248 (PMC7586992; doi:10.1111/php.13248)
Supplement: Supplementary file 1 — Figure S1. IRA irradiation causes ROS generation but it may not be involved in SG formation in NHEKs. [file PHP-96-1105-s001.pdf]

Supporting Information

Infrared-A Irradiation-induced Inhibition of Human Keratinocyte Proliferation and Potential Mechanisms

Syota Shimizu, Akihiro Aoki, Takuya Takahashi and Fumiki Harano

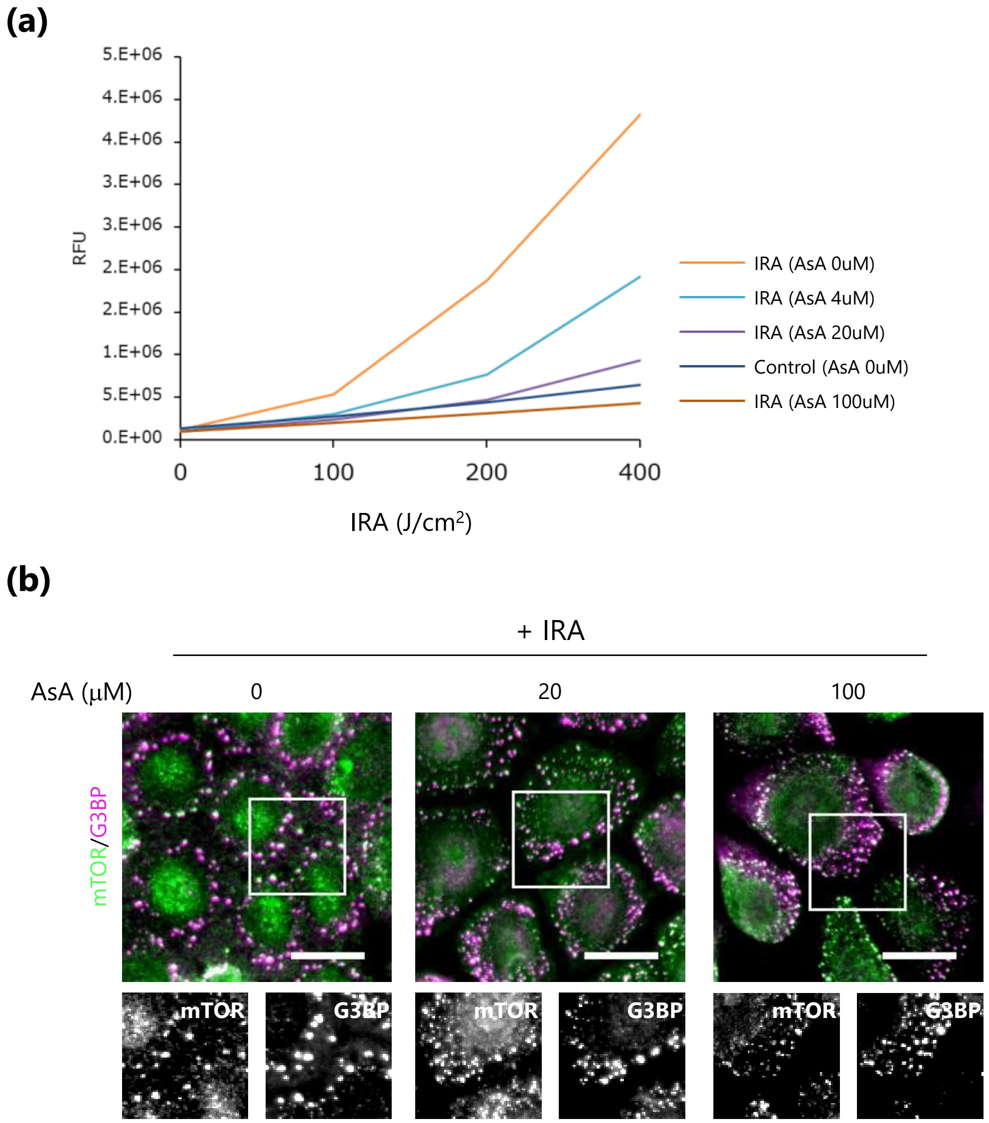

Figure S1. IRA irradiation causes ROS generation but it may not be involved in SG formation in NHEKs. (a) Intracellular ROS were detected using 2’-7’-di-hydrodichlorofluoroscein diacetate (H2DCF-DA; Molecular Probes-Invitrogen Co., Carlsbad, CA, C2938). NHEKs were treated with 5 μM H2DCF-DA for 30 min at 37°C with 5% CO_2. After loading with H2DCF-DA, the cells were washed twice with PBS (−) and then exposed to IRA (100–400 J/cm^2) with or without ascorbic acid (AsA). Representative results from two independent experiments are shown. (b) Representative immunofluorescence images of colocalization between SGs and mTOR were acquired after IRA irradiation (400 J/cm^2). Scale bar represents 20 μm.
